# Investigating the Antiproliferative and Antioxidant Properties of* Pancratium maritimum* L. (Amaryllidaceae) Stems, Flowers, Bulbs, and Fruits Extracts

**DOI:** 10.1155/2018/9301247

**Published:** 2018-09-30

**Authors:** Mariarosaria Leporini, Giorgia Catinella, Maurizio Bruno, Tiziana Falco, Rosa Tundis, Monica R. Loizzo

**Affiliations:** ^1^Department of Pharmacy, Health and Nutritional Sciences, University of Calabria, 87036 Rende (CS), Italy; ^2^Dipartimento di Scienze e Tecnologie Biologiche Chimiche e Farmaceutiche (STEBICEF), University of Palermo, Palermo, Italy

## Abstract

*Pancratium maritimum* stems, flowers, bulbs, and fruits extracts were investigated for their antiproliferative and antioxidant properties. Total phenols and total flavonoids were also determined. The* in vitro* antiproliferative activity was tested against seven cancer cell lines such as C32, HeLa, MDA-MB-231, PC3, A549, MCF-7, and LNCaP by using SRB assay. Interesting results were obtained with stems ethanol extract (ET1) against C32 cells (IC_50_ of 27.1 *μ*g/mL) and fruits aqueous extract (AQ) against MCF-7 cell line (IC_50_ of 36.5 *μ*g/mL). To define the antioxidant activity, four tests such as DPPH, ABTS FRAP, and *β*-carotene bleaching tests were used. The most promising ABTS scavenging capacity was observed with fruits ethanol extract (ET1) that showed an IC_50_ value of 6.9 *μ*g/mL. According to the correlation results, the phenols and flavonoids content could provide a fundamental contribution to the antioxidant and antiproliferative activity of* P. maritimum* extracts.

## 1. Introduction

The genus* Pancratium* (Amaryllidaceae family) comprises about 20 species.* P. maritimum *L. or marine narcissus, is a plant species typical of sandy coasts, widely disseminated from the Mediterranean to the Black Sea, including the part of the Atlantic coasts [1]. Although widely distributed,* P. maritimum *populations have declined significantly due to urbanization, tourism development, alteration and destruction of dune systems, and overharvesting [2].* P. maritimum* is used in the traditional medicine of several Mediterranean countries for its antimicrobial, antimalarial, purgative, antiviral, immune-stimulant, antalgic, anticancer, antifungal, and antioxidant properties [3–8].

Several studies focused on alkaloids as the main bioactive constituents [9, 10] while few reports investigated the nonalkaloidal composition of* P. maritimum*.

Cancer and chemoprevention represent a major challenge for health professionals worldwide [11, 12]. The pharmacological strategy, although effective in some cases, causes numerous toxic effects due to the action that these drugs also have towards healthy tissue cells that possess a high fraction of proliferating cells, such as bone marrow cells and epithelial cells [13]. The research activity in the field of oncology is therefore increasingly directed towards the selection of new molecules with greater activity and less toxicity towards healthy tissues. Therefore, the development of a safe, nontoxic plant protection product is justified [14]. Oxidative stress is closely related to all aspects of cancer, from carcinogenesis to the tumor-bearing state, from treatment to prevention [15]. Reducing oxidative stress is related to the anticancer effect. Nowak et al. [16] report the importance of chemoprevention with natural compounds to reverse, suppress, or prevent the development of invasive cancer. Moreover, natural antioxidants can eliminate free radicals such as singlet oxygen or peroxides by donating hydrogen and chelating metal ion. These activities decrease DNA damage, reduce lipid peroxidation, and inhibit cell proliferation that is closely related to cancer development [17, 18]. Hence, the studies on natural products characterized by both antioxidant and antiproliferative activities have gained increasingly greater importance. Following our previous studies, the aim of this work is to assess the* in vitro* antiproliferative activity against seven human cancer cell lines and antioxidant properties, in relation to the phenols and flavonoids content of* P. maritimum* flowers, bulbs, stems, and fruits.

## 2. Materials and Methods

### 2.1. Chemicals and Reagents

All chemicals and reagents used in this study were purchased from Sigma-Aldrich Chemical Co. Ltd (Milan, Italy) and VWR International (Milan, Italy) and, unless specified otherwise, were analytical grade or higher.

### 2.2. Plant Materials

Flowers, stems, bulbs, and fruits of* Pancratium maritimum* were collected in September 2016 in Lascari (Palermo, Italy) (38°01′48′′ N, 13°18′40′′ E, 16 m s/l) on a sandy soil. Voucher specimens (No. MB 385/16) were identified by Dr. E. Schimmenti and deposited in the Department STEBICEF, University of Palermo, Palermo, Italy.

### 2.3. Extraction Procedure

Fresh flowers, stems, bulbs, and fruits of* P. maritimum* were blended and extracted with two different methodologies: (a) sequential extraction with petroleum ether (ETP), ethanol (ET1), and water (H_2_O, 1% of H_2_SO_4_) (AQ) and (b) maceration with ethanol (ET2) (3 x 200 mL). Petroleum ether and ethanol were evaporated at low pressure, 40°C, using a Rotavapor Buchi R-200 (Buchi, Milan, Italy), whereas the water extracts were freeze-dried with Scanlaf Coolsafe 110-4.

### 2.4. Total Phenols Content

Total phenols content was evaluated by using the Folin-Ciocalteau method as previously reported [19]. A solution of Folin-Ciocalteau reagent and 15% sodium carbonate was mixed with sample. The mixture was incubated at room temperature for 2 h. The absorbance was measured at *λ* = 765 nm using a UV-Vis Jenway 6003 spectrophotometer. The total phenols content was expressed as mg chlorogenic acid equivalents/g of extract.

### 2.5. Total Flavonoids Content

Total flavonoids content was determined following the method previously described [19]. The extract was mixed with 2% aluminum chloride solution and left to incubate at room temperature for 15 min. The absorbance was measured at *λ* = 510 nm using a UV-Vis Jenway 6003 spectrophotometer. The total flavonoids content was expressed as mg quercetin equivalents/g of extract.

### 2.6. Radical Scavenging Activity

#### 2.6.1. DPPH Test

2,2-Diphenil-1-picrylhydrazyl (DPPH) radical scavenging activity was evaluated following the method previously described [20]. Different concentrations of the extract were mixed with DPPH (0.25 mM) and left to incubate at room temperature for 30 min. The absorbance was measured at *λ* = 517 nm using a UV-Vis Jenway 6003 spectrophotometer. The DPPH radicals scavenging activity was calculated as follows: DPPH scavenging activity = [(A_0_ − A_1_/A_0_) × 100], where A0 is the absorbance of the blank and A1 is the absorbance in the presence of the extract. Ascorbic acid was used as positive control.

#### 2.6.2. ABTS Assay

ABTS assay was done following the methodology previously described [21]. A solution of ABTS radical cation (ABTS^+^) and potassium persulphate was prepared. After 12 h the solution was diluted with ethanol until an absorbance of 0.70 ± 0.03 measure at *λ* = 734 nm using a UV-Vis Jenway 6003 spectrophotometer. The extract and diluted ABTS^+^ solution were mixed and after 6 min and the absorbance has been read again. The ABTS scavenging ability was calculated as follows: ABTS scavenging activity (%) = [(A_0_ − A)/A_0_] × 100 where A_0_ is the absorbance of the control reaction and A is the absorbance in the presence of extract. Ascorbic acid was used as positive control.

#### 2.6.3. *β*-Carotene Bleaching Test

The *β*-carotene bleaching test was performed following the procedure previously described [22]. A solution of *β*-carotene, linoleic acid, and 100 % Tween was prepared. After evaporation of the solvent by using a rotary evaporator the 5 mL of water was added. The emulsion was transferred into different tubes containing 0.2 mL of extract at different concentrations. The absorbance was measured at *λ* = 470 nm using a UV-Vis Jenway 6003 spectrophotometer. Propyl gallate was used as positive control.

#### 2.6.4. Relative Antioxidant Capacity Index (RACI) Calculation

The statistical application RACI was used to evaluate the antioxidant capacity of extracts [23]. The standard scores were obtained from data from different chemical methods without unrestricted units and no variance between the methods.

#### 2.6.5. Global Antioxidant Score (GAS)

The T-scores were used to calculate the value of Global Antioxidant Score (GAS). T-score is calculated by the following equation: T − score = (X − min)/(max − min), where min and max, respectively, represent the smallest and largest values of variable X among the investigated extract [24].

### 2.7. Antiproliferative Activity

#### 2.7.1. Cell Culture

Seven cancer cell lines, namely, human Caucasian breast carcinoma (MCF-7, ECACC N°:86012803), Human cervix epitheloid carcinoma (HeLa, ECACC N°:93021013), human Caucasian breast adenocarcinoma (MDA-MB-231, ECACC N°:92020424), amelanotic melanoma (C32, ATCC N°:CRL-1585), lung carcinoma A549 (ECACC No. 86012804), human Caucasian prostate carcinoma (LNCaP, ECACC N°:891102011), and human Caucasian prostate adenocarcinoma (PC3, ECACC N°: 90112714), were used in our experiments. All media, buffers, trypsin, and dyes were filter-sterilized prior to use and warmed to 37°C. The MDA-MB-231, C32, and LNCaP cells were cultured in RPMI 1640 medium, while MCF-7, HeLa, A549, and PC3 cells were cultured in DMEM. Both media were supplemented with 10% fetal bovine serum, 1% L-glutamine, and 1% penicillin/streptomycin. The cell lines were maintained at 37°C in a 5% CO2 atmosphere with 95% humidity. The cultures were passed once a week by trypsinization using a 1:30 dilution of standard Trypsin-EDTA solution. Cells counts and viability were performed using a standard trypan blue cell counting technique.

#### 2.7.2. Sulforhodamine B Assay

The antiproliferative activity was performed by using the protein-staining sulforhodamine B (SRB) assay as previously described [25]. Cells were trypsinized, counted, and placed in 96-well plates at optimal plating density of each cell line determined over a range 5-15 × 10^4^ to ensure exponential growth throughout the experimental period and to ensure a linear relationship between absorbance at 490 nm and cell number analyzed by the SRB assay and incubated to allow for cell attachment. After 24 h the cells were treated with serial dilutions of the samples. Each sample was initially dissolved in DMSO and further diluted in medium to produce different concentrations. One hundred microliters/wells of each dilution were added to the plates in six replicates to obtain the final concentrations ranging from 5 to 200 *μ*g/mL for the sample. The final mixture used for treating the cells contained not more than 0.5% of the solvent (DMSO), the same as in the solvent-control wells. After 48 h of exposure 100 *μ*L of ice-cold 40% trichloroacetic acid (TCA) was added to each well, left for 1 h at 4°C, and washed with distilled water. The TCA-fixed cells were stained for 30 min with 50 *μ*L of 0.4% (w/v) SRB in 1% acetic acid. Plates were washed with 1% HOAc and air-dried overnight. For reading plate, the bound dye was solubilised with 100 *μ*L of 10 mM tris base (tris[hydroxymethyl]aminomethane). The absorbance of each well was read on a Molecular Devices SpectraMax Plus Plate Reader (Molecular Devices, Celbio, Milan, Italy) at 490 nm. Cell survival was measured as the percentage absorbance compared to the untreated control. Vinblastine sulfate salt, doxorubicin, and taxol were used as positive control.

### 2.8. Statistical Analysis

All experiments were carried out in triplicate. Data are expressed as mean ± standard deviation (SD). The concentration giving 50% inhibition (IC_50_) was calculated by nonlinear regression with the use of Prism GraphPad Prism version 7.0 for Windows (GraphPad Software, San Diego, CA, USA). The concentration-response curve was obtained by plotting the percentage inhibition versus concentration. Differences within and between groups were evaluated by one-way analysis of variance test (ANOVA) followed by multicomparison Dunnett's test compared with the positive control.

## 3. Results and Discussion

### 3.1. Extraction Yield, Total Phenols, and Flavonoids Content

*P. maritimum* flowers, fruits, stems, and bulbs were extracted by using two methods. Firstly, plant materials were sequentially with petroleum ether (ETP), ethanol (ET1), and water (AQ). Extracts with the following yields (%) were obtained: flowers (ETP, 0.3%) (ET1, 3.6%) (AQ, 2.4%); stems (ETP, 0.1%) (ET1, 2.2%) (AQ, 0.9%); bulbs (ETP, 0.02%) (ET1, 6.5%) (AQ, 1.7%); fruits (ETP, 0.2%) (ET1, 2.5%) (AQ, 2.3%).

The second methodology consisted in the extraction of fresh and blended plant materials with ethanol (ET2) to give, after solvent evaporation, the following yields %: flowers (5.7%); stems (4.7%); bulbs (7.2%); fruits (7.0%). All samples were stored at 4°C for further investigations.

The importance to determine the content of phenols in plant extracts is related to the antioxidant capacity of these bioactive compounds that are able to act as reducing agents, free radical scavengers, metal chelators, or deactivators of singlet oxygen and/or display simultaneously more than one of these functions [26].

Table 1 showed the total phenols and total flavonoids content of different* P. maritimum* extracts. Flowers ethanol extract showed the highest total phenols content with value of 289.5 mg of chlorogenic acid equivalents/g of extract. Similar results are observed also with fruits and stems ethanol seq. extracts. Fruits ethanol seq. extract showed, also, the highest value of total flavonoids content with value of 52.71 mg of quercetin equivalents/g of extract. Recently, Johnson et al. [27] reported the total flavonoids content of* P. triflorum* extracts with values ranging from 386.66 to 4846.66 mg of GAE/g of extract for chloroform and methanol extracts, respectively.

Previously, Taie et al. [4] evaluated the total phenol and flavonoid content of* P. maritimum* root, bulb, leaves, flowers, and seeds and found the highest value in leaves (5.36 mg gallic/g extract and 1.17 mg quercetin/g extract, respectively).

### 3.2. Antiproliferative Activity

*P. maritimum* extracts were tested to evaluate their antiproliferative activity on different cancer cell lines including human Caucasian breast carcinoma (MCF-7), human cervix epithelioid carcinoma (HeLa), human Caucasian breast adenocarcinoma (MDA-MB-231), amelanotic melanoma (C32), lung carcinoma (A549), human Caucasian prostate carcinoma (LNCaP), and human Caucasian prostate adenocarcinoma (PC3). Data are reported in Table 2. All extracts showed antiproliferative effects in a concentration-dependent manner. The stems ethanol extract (ET1) was the most active against C32 cells with an IC_50_ value of 27.1 *μ*g/mL, followed by the petroleum ether extract (ETP) of bulbs (IC_50_ value of 34.7 *μ*g/mL). Both these results are of interest if compared to the positive control vinblastine with an IC_50_ value of 45.5 *μ*g/mL. The other IC_50_ values are in the range 58.4-188.6 *μ*g/mL. The stems ethanol extract (ET1) showed an activity higher than that of vinblastine (IC_50_ value 59.4* vs* 67.3 *μ*g/mL of positive control) also against lung carcinoma cells.

Except for the ETP extract, the most promising results against MCF-7 cell line were obtained with fruits extracts with IC_50_ values in the range 36.5-45.2 *μ*g/mL. Promising values were obtained with bulbs petroleum ether extract and aqueous extract that inhibited HeLa and LNCaP cells growth with IC_50_ values of 40.8 and 46.8 *μ*g/mL, respectively. Recently, Tayoub et al. [28] evaluated the effects of Iranian* P. maritimum* bulbs, leaves, flowers, and roots on human breast cancer cells MDA-MB-321. For this purpose plant material was extracted by maceration with ethanol. The antiproliferative activity was assessed using BD biosciences cell viability kit with exposure time of 24, 48, 72, and 96 hours of exposure. As in our experiments all extract inhibited cancer cell in a dose-dependent manner however a more pronounced cell growth inhibitory activity was observed also in dependence of the time. Generally, bulbs showed more antiproliferative activities than leaf extract. Bulbs ethanol extract showed the most promising activity after 48 h of exposure with IC_50_ value of 0.039 mg/mL. The cytotoxic activity is mediated by cell cycle cell arrest at S and G2/M phases. The expression of cyclin B1, Bcl-2, and Ki67 was also affected by plant extracts.

Based on the indications of the National Cancer Institute, plant extracts with an IC_50_ value less of 30 *μ*g/ml are to be considered as promising anticancer agents that needed further investigation [29]. In this preliminary study, the focus of our interest was on* P. maritimum* crude extracts. Further studies will be done in order to identify phytochemicals responsible of the activity and their mechanism of action.

### 3.3. Antioxidant Activity

Despite the presence of the several antioxidant defence systems to neutralize oxidative stress, oxidative damage may occur to cell structure and may induce somatic mutations and neoplastic transformation. Indeed, cancer initiation and progression has been linked to oxidative stress by inducing DNA damage, increasing DNA mutations, and cell proliferation [30]. Counteracting oxidative stress with potent antioxidant agents is a very active field of research. Herein, the antioxidant activity of* P. maritimum* extracts was examined using different* in vitro* methods. All samples showed concentration-dependent antioxidants effects. Data are reported in Table 3.

The most promising scavenging capacity was observed with fruits ethanol extract (ET1) that inhibited ABTS radicals with an IC_50_ value of 6.9 *μ*g/mL, followed by flowers petroleum ether extract that showed an IC_50_ value of 32.2 *μ*g/mL in DPPH assay. A significant protection of lipid peroxidation was observed with flowers aqueous extract that showed IC_50_ values of 8.2 and 7.2 *μ*g/mL, after 30 and 60 minutes of incubation, respectively. A moderate ferric reducing activity for all tested samples was observed.

Previously, Nikolova et al. [31] found an IC_50_ value greater than 200 *μ*g/mL for the* P. maritimum* methanol bulbs extract. A promising DPPH and ABTS radical scavenging activity was observed also with Egyptian* P. maritimum* flowers and leaves methanol extracts [4]. In particular flowers and leaves extracts recorded the highest DPPH radical scavenging potential with percentage of 85.2 and 81.3%, respectively. Moreover, flowers significantly inhibited ABTS^+·^ with percentage of 72.3%. The leaves antioxidant potential was confirmed, also in Tunisian* P. maritimum* [32]. Leaves extract showed stronger ORAC and DPPH inhibition compared to bulbs extract. The comparison of diethyl ether and ethyl acetate fractions of the aqueous extract of* P. foetidum* leaves confirmed that the DPPH radical scavenging potential is related to the total phenols content [33]. The key role of phenols content in antioxidant capacity, with particular reference to the free radical scavenging activity, was previously evidenced by Elmastas et al. [34].

In our study, a positive correlation was found with total phenols content and DPPH, *β*-carotene after 30 and 60 minutes of incubation, and FRAP test. In addition, a positive correlation between total flavonoids content and DPPH and FRAP test was observed. The Relative Antioxidant Capacity Index (RACI) and the Global Antioxidant Score (GAS) are calculated and values are comprised in the range 0.56-38 and 0.02-3.50, respectively.

## 4. Conclusions

In this study, we investigated the total phenols and flavonoids content of* P. maritimum* stems, flowers, bulbs, and fruits extracts and their antiproliferative and antioxidant properties. The antiproliferative effect of the ethanol extract of stems against C32 and A-549 cells may be related to their antioxidant activity. Moreover, the ethanol extract of fruits, with the higher content of flavonoids, presents the highest radical scavenging activity in ABTS test. In conclusion, the results revealed that* P. maritimum* extracts can provide a good source of antioxidant compounds and showed significant antiproliferative effects.

## Figures and Tables

**Table 1:** Total phenols and total flavonoids content of* P. maritimum* extracts.

Sample	Total Phenols Content^a^	Total Flavonoids Content^b^
Flowers		

ETP	242.2 ± 2.8	39.8 ± 0.1
ET1	228.6 ± 2.4	45.7 ± 0.2
AQ	40.4 ± 1.1	26.9 ± 0.2
ET2	289.5 ± 2.6	41.1 ± 0.5

Fruits		

ETP	30.1 ± 1.7	21.7 ± 0.2
ET1	277.8 ± 2.9	52.7 ± 0.3
AQ	30.9 ± 0.8	10.3 ± 0.4
ET2	72.7 ± 1.5	30.3 ± 0.3

Stems		

ETP	246.7 ± 2.3	30.7 ± 0.1
ET1	260.0 ± 3.2	35.2 ± 0.1
AQ	246.7 ± 3.3	30.6 ± 0.9
ET2	35.1 ± 1.2	14.4 ± 0.2

Bulbs		

ETP	213.8 ± 3.5	30.6 ± 0.1
ET1	48.4 ± 1.5	24.4 ± 0.3
AQ	30.0 ± 0.9	17.1 ± 0.2
ET2	60.9 ± 1.3	24.6 ± 0.4

^1^Data are expressed as mean ± SD (*n*= 3). ETP: petroleum ether extract; ET1: sequential extraction with ethanol; AQ: sequential extraction with water; ET2: maceration with ethanol; ^a^mg of chlorogenic acid equivalents/g of extract. ^b^mg of quercetin equivalents/g of extract.

**Table 2:** Antiproliferative capacity [IC_50_ (*μ*g/mL)] of *P. maritimum* extracts.

*P. maritimum *	MCF-7	HeLa	MDA-MB-231	C32	A549	LNCaP	PC3
Flowers							

ETP	156.3 ± 6.1^*∗∗∗*^	175.1 ± 5.4^*∗∗∗*^	NA	89.5 ± 3.8^*∗∗∗*^	100.2 ± 3.7^*∗∗∗*^	98.4 ± 4.4^*∗∗∗*^	171.5 ± 5.7^*∗∗∗*^
ET1	78.7 ± 3.4^*∗∗∗*^	80.8 ± 3.9^*∗∗∗*^	134.6 ± 4.3^*∗∗∗*^	67.2 ± 2.9^*∗∗∗*^	81.3 ± 3.1^*∗∗∗*^	75.8 ± 3.5^*∗∗∗*^	119.6 ± 4.0^*∗∗∗*^
AQ	141.7 ± 4.3^*∗∗∗*^	134.7 ± 3.8^*∗∗∗*^	NA	179.8 ± 5.4^*∗∗∗*^	112.2 ± 3.8^*∗∗∗*^	85.5 ± 3.5^*∗∗∗*^	176.7 ± 3.9^*∗∗∗*^
ET2	176.2 ± 4.3^*∗∗∗*^	198.7 ± 3.5^*∗∗∗*^	NA	172.1 ± 2.8^*∗∗∗*^	100.1 ± 1.8^*∗∗∗*^	85.5 ± 3.5^*∗∗∗*^	176.7 ± 3.9^*∗∗∗*^

Fruits							

ETP	171.6 ± 5.3^*∗∗∗*^	132.9 ± 3.3^*∗∗∗*^	NA	79.2 ± 3.4^*∗∗∗*^	123.1 ± 3.7^*∗∗∗*^	79.6 ± 3.2^*∗∗∗*^	143.7 ± 3.8^*∗∗∗*^
ET1	45.2 ± 3.1^*∗∗∗*^	36.9 ± 3.4^*∗∗∗*^	144.9 ± 4.4^*∗∗∗*^	61.0 ± 3.9^*∗∗∗*^	80.5 ± 3.9^*∗∗∗*^	77.9 ± 3.7^*∗∗∗*^	121.3 ± 4.4^*∗∗∗*^
AQ	36.5 ± 3.7^*∗∗∗*^	49.9 ± 3.2^*∗∗∗*^	185.4 ± 2.9^*∗∗∗*^	65.2 ± 3.7^*∗∗∗*^	82.6 ± 4.0^*∗∗∗*^	65.3 ± 3.8^*∗∗∗*^	128.4 ± 4.6^*∗∗∗*^
ET2	44.3 ± 3.9^*∗∗∗*^	42.7 ± 3.8^*∗∗∗*^	155.7 ± 3.9^*∗∗∗*^	58.4 ± 3.8^*∗∗∗*^	122.7 ± 4.6^*∗∗∗*^	46.8 ± 4.2^*∗∗∗*^	110.3 ± 2.7^*∗∗∗*^

Stems							

ETP	122.8 ± 5.8^*∗∗∗*^	135.2 ± 6.1^*∗∗∗*^	NA	71.1 ± 3.0^*∗∗∗*^	85.3 ± 3.5^*∗∗∗*^	70.6 ± 3.4^*∗∗∗*^	129.9 ± 4.8^*∗∗∗*^
ET1	56.2 ± 3.4^*∗∗∗*^	50.1 ± 3.2^*∗∗∗*^	NA	27.1 ± 3.5^*∗∗∗*^	59.4 ± 3.5^*∗∗∗*^	70.1 ± 3.2^*∗∗∗*^	178.2 ± 3.3^*∗∗∗*^
AQ	132.1 ± 2.2^*∗∗∗*^	NA	176.3 ± 5.6^*∗∗∗*^	188.6 ± 3.4^*∗∗∗*^	192.1 ± 4.9^*∗∗∗*^	88.2 ± 3.1^*∗∗∗*^	100.3 ± 2.6^*∗∗∗*^
ET2	183.5 ± 5.4^*∗∗∗*^	NA	NA	187.2 ± 3.5^*∗∗∗*^	123.4 ± 5.9^*∗∗∗*^	178.9 ± 4.3^*∗∗∗*^	173.9 ± 5.1^*∗∗∗*^

Bulbs							

ETP	45.9 ± 3.2^*∗∗∗*^	40.8 ± 4.9^*∗∗∗*^	111.7 ± 4.1^*∗∗∗*^	34.7 ± 3.9^*∗∗∗*^	76.3 ± 3.6^*∗∗∗*^	66.1 ± 3.2^*∗∗∗*^	80.2 ± 4.0^*∗∗∗*^
ET1	NA	186.5 ± 5.48^*∗∗∗*^	NA	125.7 ± 4.8^*∗∗∗*^	187.1 ± 4.7^*∗∗∗*^	167.2 ± 5.4^*∗∗∗*^	179.1 ± 5.5^*∗∗∗*^
AQ	182.5 ± 5.4^*∗∗∗*^	178.9 ± 4.3^*∗∗∗*^	NA	182.9 ± 3.9^*∗∗∗*^	182.5 ± 5.4^*∗∗∗*^	178.9 ± 4.3^*∗∗∗*^	176.3 ± 5.6^*∗∗∗*^
ET2	138.6 ± 2.2^*∗∗∗*^	NA	144.2 ± 2.8^*∗∗∗*^	181.2 ± 3.6^*∗∗∗*^	190.3 ± 4.1^*∗∗∗*^	75.3 ± 3.3^*∗∗∗*^	102.3 ± 2.8^*∗∗∗*^

Positive control							

Vinblastine				45.5 ± 1.9	67.3 ± 2.0	29.3 ± 0.9	
Doxorubicin	4.04 ± 0.4	3.6 ± 0.3	14.9 ± 1.4				1.5 ± 0.1
Taxol	0.08 ± 0.006	0.011 ± 0.008	8.62 ± 0.6				2.3 ± 0.4

Data are expressed as mean ± standard deviation (SD) (n= 3). ETP: petroleum ether extract; ET1: sequential extraction with ethanol; AQ: sequential extraction with water; ET2: maceration with ethanol; MCF-7: human Caucasian breast carcinoma HeLa: Human cervix epithelioid carcinoma; MDA-MB-231: human Caucasian breast adenocarcinoma; C32: amelanotic melanoma; A549: lung carcinoma; LNCaP: human Caucasian prostate carcinoma; PC3: human Caucasian prostate adenocarcinoma. MCF-7, HeLa, and MDA-MB-231: one-way ANOVA ^*∗∗∗*^p <0.0001 followed by multicomparison Dunnett's test: ^*∗∗∗*^p <0.01 compared with doxorubicin. C32, A549, and LNCaP: one-way ANOVA ^*∗∗∗*^p <0.0001 followed by multicomparison Dunnett's test: ^*∗∗∗*^p <0.01 compared with vinblastine.

**Table 3:** Antioxidant activity of *P. maritimum* extracts.

*P. maritimum *	DPPH test (IC_50_ *μ*g/mL)	ABTS test (IC_50_ *μ*g/mL)	*β*-carotene bleaching test (IC_50_ *μ*g/mL)	*β*-carotene bleaching test (IC_50_ *μ*g/mL)	FRAP test *μ*M Fe (II)/g	RACI	GAS
			30 min	60 min			

Flowers							

ETP	32.2 ± 3.2^*∗∗∗*^	16.7 ± 1.6^*∗∗∗*^	88.7 ± 8.1^*∗∗∗*^	72.5 ± 7.1^*∗∗∗*^	3.8 ± 0.4^*∗∗∗*^	0.05	1.93
ET1	81.3 ± 8.1^*∗∗∗*^	10.4 ± 1.2^*∗∗∗*^	9.2 ± 0.9^*∗∗∗*^	19.4 ± 2.0^*∗∗∗*^	15.6 ± 1.2^*∗∗∗*^	-0.22	1.18
AQ	49.7 ± 4.7^*∗∗∗*^	14.4 ± 1.4^*∗∗∗*^	8.2 ± 8.1^*∗∗∗*^	7.2 ± 1.5^*∗∗∗*^	NA	-0.31	0.24
ET2	95.3 ± 9.1^*∗∗∗*^	11.6 ± 1.1^*∗∗∗*^	63.6 ± 6.3^*∗∗∗*^	64.1 ± 6.2^*∗∗∗*^	10.1 ± 1.0^*∗∗∗*^	0.15	2.26

Fruits							

ETP	35%	13.3 ± 1.3^*∗∗∗*^	48.2%	37.7%	NA	-0.38	0.02
ET1	846.1 ± 44.2^*∗∗∗*^	6.9 ± 0.6^*∗∗∗*^	63.3 ± 6.1^*∗∗∗*^	77.5 ± 7.1^*∗∗∗*^	11.8 ± 1.1^*∗∗∗*^	0.56	2.96
AQ	40.4%	98.2 ± 9.6^*∗∗∗*^	11.2 ± 1.3^*∗∗∗*^	51.1 ± 5.1^*∗∗∗*^	2.4 ± 0.3^*∗∗∗*^	0.04	1.95
ET2	746.8 ± 48.2^*∗∗∗*^	10.0 ± 1.0^*∗∗∗*^	94.9 ± 9.2^*∗∗∗*^	80.9 ± 8.1^*∗∗∗*^	1.8 ± 0.1^*∗∗∗*^	-0.16	0.92

Stems							

ETP	711.5 ± 23.5^*∗∗∗*^	23.4 ± 2.4^*∗∗∗*^	57.8 ± 5.9^*∗∗∗*^	89.1 ± 8.7^*∗∗∗*^	5.1 ± 0.5^*∗∗∗*^	0.54	2.60
ET1	84.1 ± 8.4^*∗∗∗*^	8.1 ± 0.8^*∗∗∗*^	81.6 ± 8.2^*∗∗∗*^	98.0 ± 9.4^*∗∗∗*^	19.7 ± 1.4^*∗∗∗*^	0.23	2.94
AQ	38.5%	110.9 ± 15.3^*∗∗∗*^	11.4 ± 1.2^*∗∗∗*^	40.5 ± 4.0^*∗∗∗*^	NA	-0.09	1.10
ET2	105.7 ± 15.2^*∗∗∗*^	16.4 ± 1.4^*∗∗∗*^	95.1 ± 9.1^*∗∗∗*^	81.6 ± 8.2^*∗∗∗*^	5.6 ± 0.6^*∗∗∗*^	-0.33	0.19

Bulbs							

ETP	923.0 ± 50.6^*∗∗∗*^	272.4 ± 16.5^*∗∗∗*^	84.2 ± 8.1^*∗∗∗*^	13.2 ± 1.1^*∗∗∗*^	NA	0.24	3.01
ET1	46%	8.9 ± 0.9^*∗∗∗*^	50.2 ± 48.2^*∗∗∗*^	9.9 ± 0.9^*∗∗∗*^	NA	-0.32	0.63
AQ	149.3 ± 14.2^*∗∗∗*^	231.8 ± 22.6^*∗∗∗*^	21.5 ± 2.3^*∗∗∗*^	23.9 ± 2.4^*∗∗∗*^	4.2 ± 0.3^*∗∗∗*^	-0.15	1.69
ET2	42.2%	12.0 ± 1.1^*∗∗∗*^	9.4 ± 8.7^*∗∗∗*^	8.0 ± 7.1^*∗∗∗*^	NA	0.52	3.50

Positive control							

Ascorbic acid	5.0 ± 0.8	1.7 ± 0.06					
Propyl gallate			0.09 ± 0.004	0.09 ± 0.004			
BHT					63.2 ± 4.3		

Data are expressed as mean ± standard deviation (SD) (n= 3). ETP: petroleum ether extract; ET1: sequential extraction with ethanol; AQ: sequential extraction with water; ET2: maceration with ethanol; RACI: Relative Antioxidant Capacity Index; GAS: Global Antioxidant Score. DPPH Radical Scavenging Activity Assay: one-way ANOVA ^*∗∗∗*^p <0.0001 followed by multicomparison Dunnett's test: ^*∗∗∗*^p <0.01 compared with ascorbic acid. ABTS test: one-way ANOVA ^*∗∗∗*^p <0.0001 followed by a multicomparison Dunnett's test: ^*∗∗∗*^p <0.01 compared with ascorbic acid. *β*-carotene bleaching test at 30 minutes of incubation: one-way ANOVA ^*∗∗∗*^p <0.0001 followed by multicomparison Dunnett's test: ^*∗∗∗*^p <0.01 compared with propyl gallate. *β*-carotene bleaching test at 60 minutes of incubation: one-way ANOVA ^*∗∗∗*^p <0.0001 followed by multicomparison Dunnett's test: ^*∗∗∗*^p <0.01 compared with propyl gallate. Ferric Reducing Antioxidant Power (FRAP): one-way ANOVA ^*∗∗∗*^p <0.0001 followed by multicomparison Dunnett's test ^*∗∗*^p <0.01 compared with BHT.

## Data Availability

The data used to support the findings of this study are available from the corresponding author upon request.

## Abbreviations

ABTS: 2,2′-Azinobis (3-ethylbenzothiazoline-6-sulfonic acid) diammonium salt
DMSO: Dimethyl sulfoxide
DPPH: 2,2-Diphenyl-1-picrylhydrazyl
FRAP: Ferric Reducing Ability Power
GAS: Global Antioxidant Score
IC_50_: Concentration giving 50% inhibition
RACI: Relative Antioxidant Capacity Index
ROS: Reactive Oxygen Species
SD: Standard deviation
SRB: Sulforhodamine B
TCA: Trichloroacetic acid.
